# Syrian hamsters (*Mesocricetus auratus*) as an upper respiratory tract model for respiratory syncytial virus infection

**DOI:** 10.1038/s44298-024-00086-6

**Published:** 2025-01-08

**Authors:** Sophie M. Kolbe, Kate Guilfoyle, Wencke Reineking, Geert van Amerongen, Guido van der Net, Sandra Lockow, Wolfgang Baumgärtner, Martin Ludlow, Albert D.M.E Osterhaus

**Affiliations:** 1 Research Center for Emerging Infections and Zoonoses, University of Veterinary Medicine Hannover, Hannover, Germany; 2 Preclinical Specialty Services, Cerba Research, Schaijk, The Netherlands; 3 Department of Pathology, University of Veterinary Medicine, Foundation, Hannover, Germany

**Keywords:** Virology, Immunohistochemistry

## Abstract

Respiratory syncytial virus (RSV) is one of the leading causes of respiratory tract infection in children, immunocompromised individuals and older adults. Vaccines have recently been approved for use in adults and although further efforts to develop suitable interventions for children are ongoing, there are limited animal models for RSV infection. For preclinical efficacy testing of prophylactic and therapeutic treatments cotton rat and ferret models can be used. However, these can be expensive, difficult to source and house, and often have limitations such as insufficient virus replication in the respiratory tract and/or lack of horizontal transmission. In this study, Syrian hamsters (*Mesocricetus auratus)*, which are relatively cheap, easy to source and house, were inoculated intranasally with a recombinant RSV-A-0594 strain expressing EGFP and using virological and pathological analyses. Viral replication was assessed and compared to viral replication in the ferret model. Although there was limited virus infection of the lower respiratory tract of Syrian hamsters, we show that a contemporary recombinant RSV-A strain replicates efficiently in the upper respiratory tract of Syrian hamsters (titers up to 4.5 Log10 TCID50/g and 12 Log10 RNA copies/g). These titers are comparable to those found in the ferret upper respiratory tract tissues post-infection with the same virus strain (up to 6.0 Log10 TCID50/g and 12 Log 10 RNA copies/g). Fluorescent regions indicating virus infection were macroscopically visible under UV-light in the nasal turbinates and histological assessment showed mucosal inflammation with necrotic cells in this tissue. In summary, Syrian hamsters generally displayed less severe systemic and pulmonary changes than ferrets, but do appear to be a promising model for upper respiratory tract infection with RSV.

## Introduction

Respiratory syncytial virus (RSV) of the genus *Orthopneumovirus* in the family *Pneumoviridae* is one of the leading causes of respiratory tract infections in children under five years of age^1^. Re-infections occur throughout life with RSV-infected adults displaying mostly mild symptoms associated with upper respiratory tract infection (URTI). However, RSV can also cause more severe diseases involving lower respiratory tract infections (LRTI) in older adults and immunocompromised individuals. These groups are of particular risk of RSV infection upon contact with symptomatic infected children^2–5^. Two subtypes co-circulate during the year with pandemic peaks during the winter months^6^. There are currently two FDA- and EMA-approved human monoclonal antibodies (HuMAbs) (Palivizumab and Nirsevimab) and three FDA- and EMA-approved vaccines against RSV^7,8^. The HuMAbs are used preventively to protect newborn babies, whereas the vaccines have thus far only been approved for use in adults^7–10^.

Despite the discovery of RSV over 65 years ago, efficacious vaccines were only approved in 2023. Although there have been several reasons underlying this extended time, a lack of animal models that fully recapitulate the pathogenesis of RSV infection in humans has complicated the testing of vaccine candidates. The most used animal models for RSV in pre-clinical testing of potential therapeutics or vaccines are cotton rats, mice, and more recently ferrets. RSV-infected cotton-rats do not develop clinical signs and do not transmit the virus to contact animals. Upon intranasal (IN) infection, RSV replicates in the nose and lungs of cotton rats^11,12^. Upon IN-infection of ferrets, RSV is readily detected in the nasal passages, but virus replication in the lung is only observed in infant ferrets^12–14^. However, intratracheal (IT) infection of ferrets of all age groups with a low passage contemporary virus strain does result in LRTI^15^. In hamsters, laboratory-adapted and older RSV-A strains have been previously reported to cause virus replication with limited respiratory tract infection in the absence of overt macroscopic and histological evidence of pulmonary pathology^16^. Nevertheless, there would be several practical advantages of using the hamsters as an alternative to other small animal models. Hamsters are relatively affordable, easy to source and to handle.

In the present study, ferrets and hamsters were infected with a recombinant (r) RSV strain derived from the contemporary ON1 genotype (RSV-A-0594)^17^. For better visualization, an enhanced green fluorescent protein (EGFP) ORF was integrated into the genomic structure of the recombinant virus. We have performed virological and pathological analyses of swabs and tissue samples, to show that hamsters can be useful as a model for RSV-A URTI.

## Results

### Body weight

As the aim of the study was to evaluate Syrian hamsters as an upper respiratory tract model for RSV infection, hamsters and ferrets were inoculated with a recombinant (r)RSV-A-0594 strain expressing EGFP. Body weight was recorded daily and compared to pre-infection body weight. After infection, the weight of the ferrets decreased by 1.8% in the group euthanized 4 dpi and by 6.4% in the group euthanized 6 dpi. In contrast, the body weight of the hamsters increased by around 4 to 4.7% at each time point (Fig. S2).

### Virus titer and copy numbers in infected animals

**Fig. 1:**
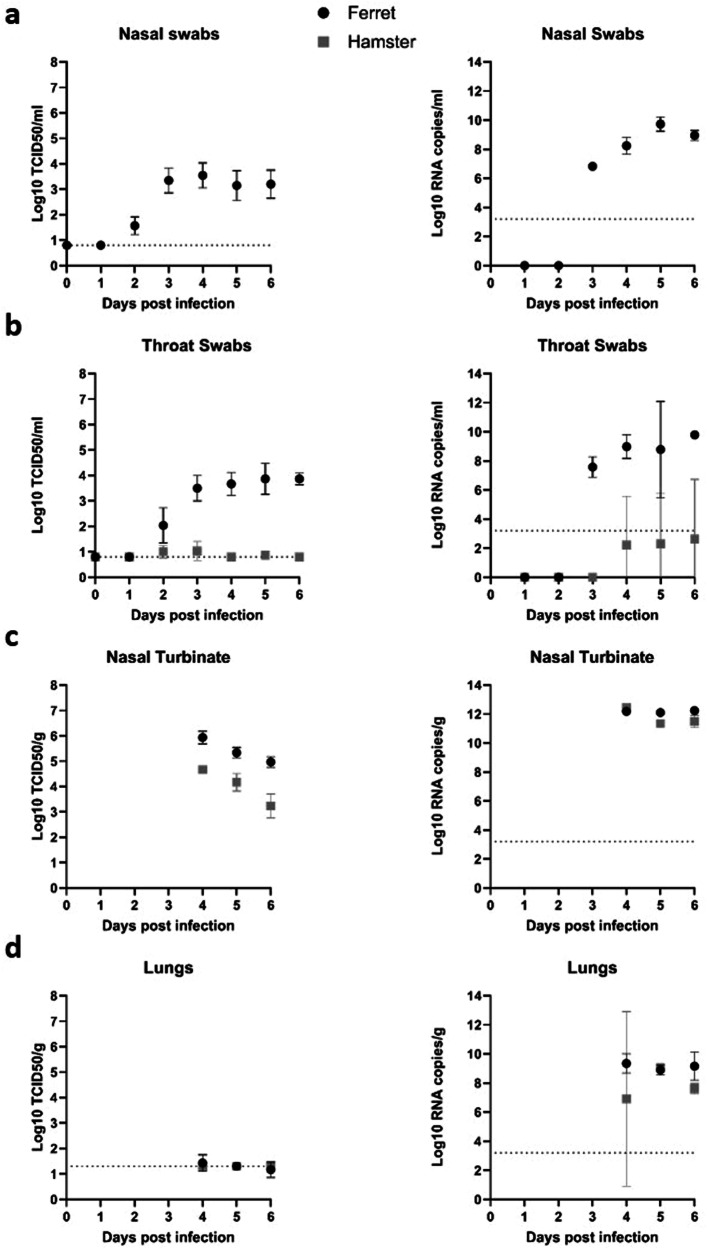
Virus titer and genome copy number analyses of swab and tissue samples obtained from rRSV-A-0594-eGFP infected hamsters and ferrets. RSV titer and copy number in (**a**) nasal swabs collected from infected ferrets (day 1 to 6 pi). **b** Throat swabs collected from ferrets and hamster (days 1 to 6 pi). **c** Nasal turbinate tissue and (**d**) Lung tissue collected at time of euthanasia (day 4 to 6 pi). The limit of detection (LOD) for the virus titers of the swabs is 0.8 log10 TCID_50_/mL and for the tissues, LOD varies as individual tissue weights are used for virus titer calculation but is mostly in the area of 1.3 log10 TCID_50_/mL. The LOD for the RSV genome copy number is a 3.2 Log10 RNA copies/ml. Error bars show standard deviation.

Daily nasal swabs were collected from the ferrets and daily throat swabs were collected from the ferrets and the hamsters. After euthanasia (days 4 to 6 pi), tissues were collected. To evaluate virus replication and spread in the models, virological (swabs and tissues) and pathological (tissues) analyses were performed. Titration of the throat and nasal swabs show there were detectable infectivity titers in all samples collected from the ferrets from day 2 pi to day 6 pi. Group mean titers increased from day 2 to 3 pi, and then ranged between 3 to 4 log_10_ TCID_50_/mL on days 3 to 6 pi. There were no detectable infectivity titers in most hamster throat swabs. However, infectivity titers were detected in some hamsters on days 2 to 5 pi, ranging between 0.8 and 1.9 log_10_ TCID_50_/mL (Fig. 1a, b).

Viral RNA copy numbers were calculated as described in the Supplementary Appendix and all samples with a Ct value > 35 were excluded. Viral RNA levels detected in the swabs of the ferrets correlated with the infectivity titers. In contrast to the infectivity titers, the viral RNA copy numbers in the throat swabs of the hamsters increased after day 2 pi and were at detectable levels in all samples until day 6 pi. However, it remained below those of the ferrets (Fig. 1a, b).

Infectivity titers and copy numbers were detected in all nasal turbinate tissues of both species collected on days 4, 5, and 6 pi. Peak group mean infectivity titer was detected in both species on day 4 pi, 5.9, and 4.7 log_10_ TCID_50_/g in the ferrets and hamsters respectively. Peak copy number in the nasal turbinate was detected at day 6 pi for the ferrets with a mean copy number of 12.2 Log_10_ RNA copies/ g tissue and at day 4 pi for hamsters with a mean copy number of 12.4 Log_10_ RNA copies/ g tissue, whereas the virus titers in the lungs tissue of the ferrets and hamster stay under 1 log_10_ TCID_50_/g. The Log_10_ RNA copies/g lung tissue of the ferrets and hamsters peak at day 4 pi for the ferrets with a mean copy number of 9.3 Log_10_ RNA copies/ g tissue and at day 5 pi for the hamsters with a mean copy number of 9 Log_10_ RNA copies/ g tissue (Fig. 1c, d).

### Pathology

**Fig. 2:**
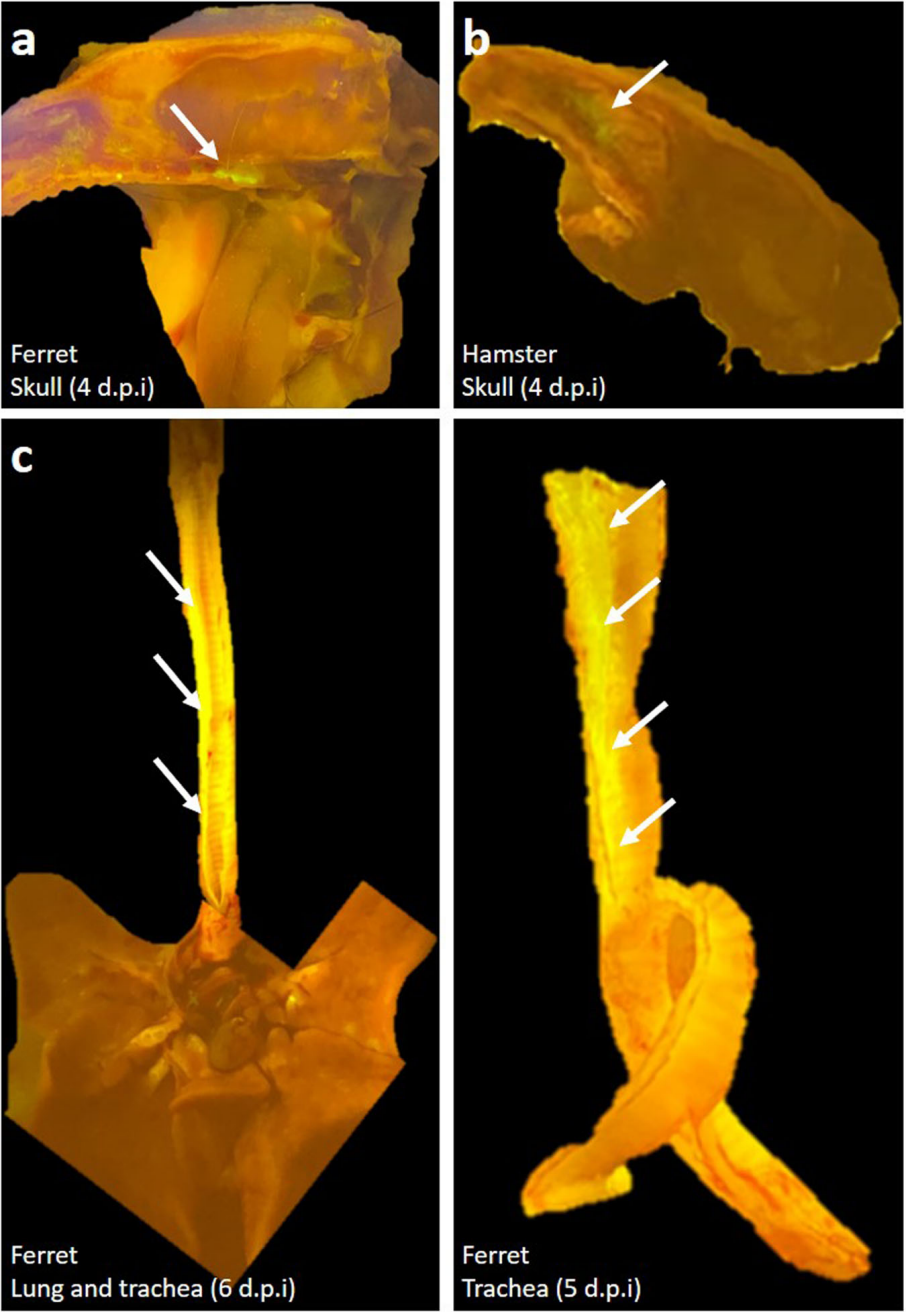
Fluorescent images taken between day 4 to 6 p.i. Images show a longitudinal cross-section of the ferret skull (**a**), hamster skull (**b**) and ferret lung and trachea day 6 and ferret trachea day 5 (**c**) post infection with rRSV-A-0594-EGFP. Images were taken between day 4 and 6 pi. and specific areas of EGFP fluorescence are highlighted with an arrow.

**Fig. 3:**
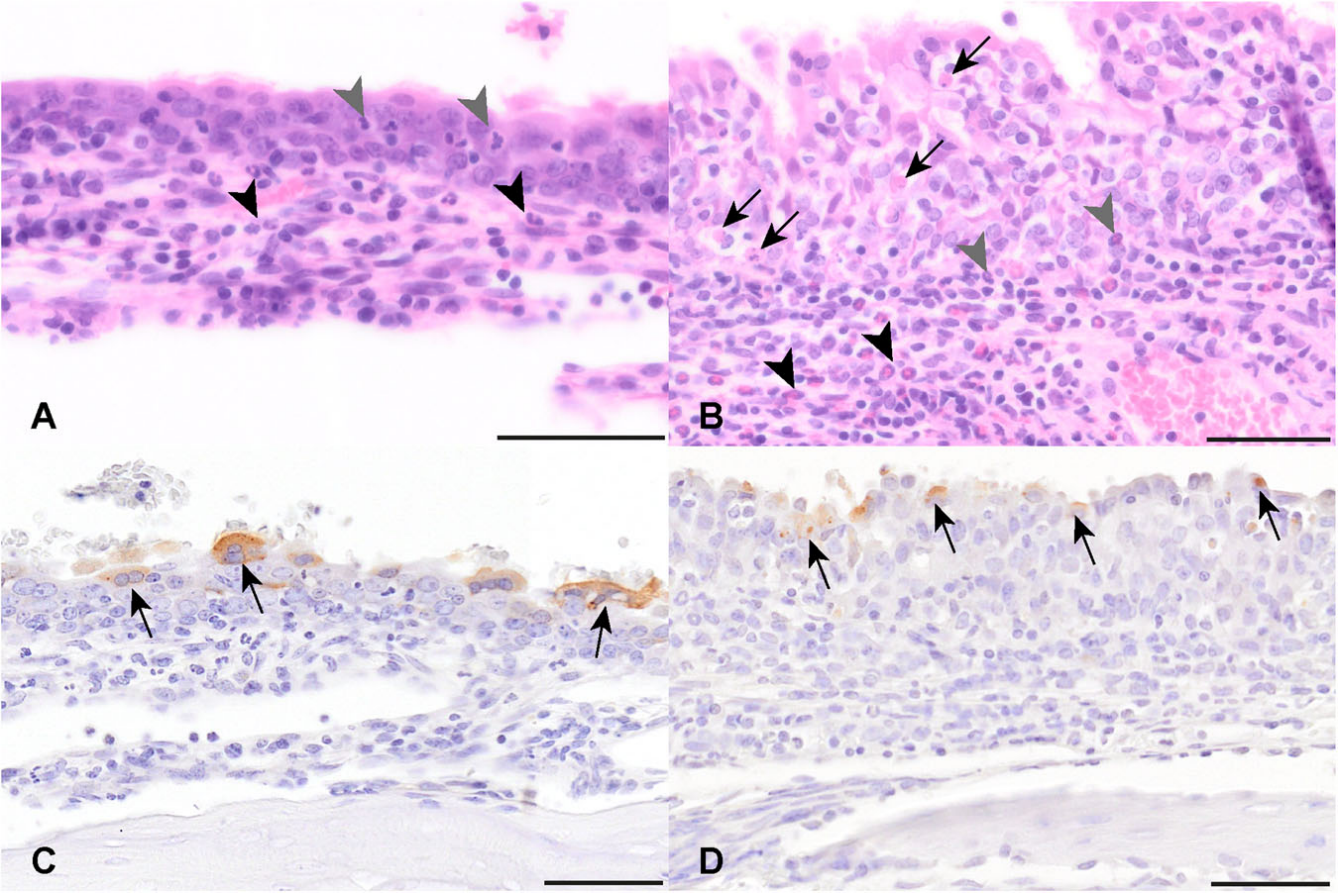
RSV infection of nasal respiratory mucosa. **A**, **B** Within the respiratory mucosa of ferrets (**a**, nasal turbinates, H&E) and hamsters, (nasal cavity, H&E) a multifocal infiltration of intra-epithelial (grey arrowheads) and submucosal granulocytes (black arrowheads) is present. In the hamster, there are also low to moderate numbers of necrotic single cells (arrows). Multifocal, epithelial cells in the nasal turbinates of ferrets (**C**) and nasal cavity of hamsters (**D**) are immunolabeled for RSV antigen (arrow) by chromogenic DAB immunochemistry. Bars, 50 μm.

To assess the extent of viral infection in the respiratory tract, tissues were assessed under ultraviolet light to illuminate longitudinal cross-sections of the skulls, lungs, and trachea of ferrets and hamsters following euthanasia at 4–6 days post-infection with rRSV-A-EGFP. Green-fluorescent cells indicative of virus infection were detectable in some regions of the skull, lungs, and trachea (Fig. 2). Histological analysis of tissue sections from both, infected hamsters and ferrets, showed multifocal infiltration of mainly granulocytes as well as macrophages and lymphocytes within the nasal respiratory mucosa. Inflammatory infiltrates were mainly present within the submucosa, but also extended into the respiratory epithelium. There were multiple necrotic cells throughout the respiratory mucosa, which was detected to a lesser extent in ferrets in comparison to hamsters. The nasal respiratory mucosa of the hamster also showed multifocal hyperplasia (Fig. 3A, B). Variable amounts of intraluminal cellular debris and granulocytes were detectable in the nasal cavity of both species. There was a minimal infiltration of granulocytes within the olfactory submucosa often accompanied by granulocytic pavementing characterized by alignment of granulocytes along the luminal surface of endothelial cells in submucosal vessels. In ferrets and hamsters, the rhinitis score ranged between two and three. Five ferrets and four hamsters had a rhinitis score of two and four ferrets/hamsters had a score of three. For hamster 18, no nasal tissues were available. Immunoreactivity for RSV antigen ranged between zero and two in the respiratory mucosa and zero and one for olfactory mucosa in ferrets and hamsters. Staining intensity was lower in hamster tissues (Fig. 3C, D). All ferrets showed RSV antigen labeling in the respiratory mucosa, with five animals having a score of 1 and two animals having a score of 2. In the hamsters, six animals showed RSV immunolabeling, out of which 5 had a score of 1 and one had a score of 2. Two animals did not show any immunoreactivity with the applied antibody. A mild multifocal immunoreactivity was detected in the olfactory mucosa of three ferrets and five hamsters, the remaining animals did not show immunolabeling. The six hamsters with respiratory mucosal immunoreactivity also showed weak multifocal immunolabeling in the olfactory mucosa (score 1).

**Fig. 4:**
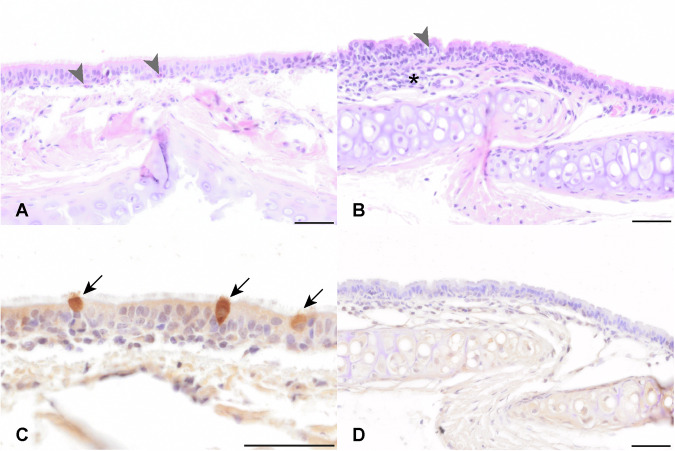
RSV infection of tracheal mucosa. **A** Within, scattered granulocytes are present within the epithelium (grey arrowheads) of the tracheal mucosa of ferrets. H&E; (**B**) A multifocal infiltration of intra-epithelial granulocytes (grey arrowheads) and submucosal mononuclear cells (asterisk) is present within the hamster trachea epithelium. H&E; **C** Multifocal, epithelial cells in the ferret mucosa are immunolabeled for RSV antigen (arrows) by chromogenic DAB immunochemistry. **D** No immunolabeling for RSV antigen is present in the hamster trachea. Bars, 50 μm.

Tracheal infiltration was mainly composed of submucosal lymphocytes and macrophages admixed with occasional granulocytes (Fig. 4A, B). Seven ferrets had a tracheal histopathological score of 1 and one ferret each had a score of 2 and 3. In four ferrets, a RSV immunohistochemistry (IHC) score of 2 was noted and in one ferret a score of 1. The remaining four ferret tracheas did not show immunolabeling for RSV antigen. Three hamsters each had a score of 1, 2 and 3, but did not show any immunolabeling for RSV antigen (Fig. 4C, D).

**Fig. 5:**
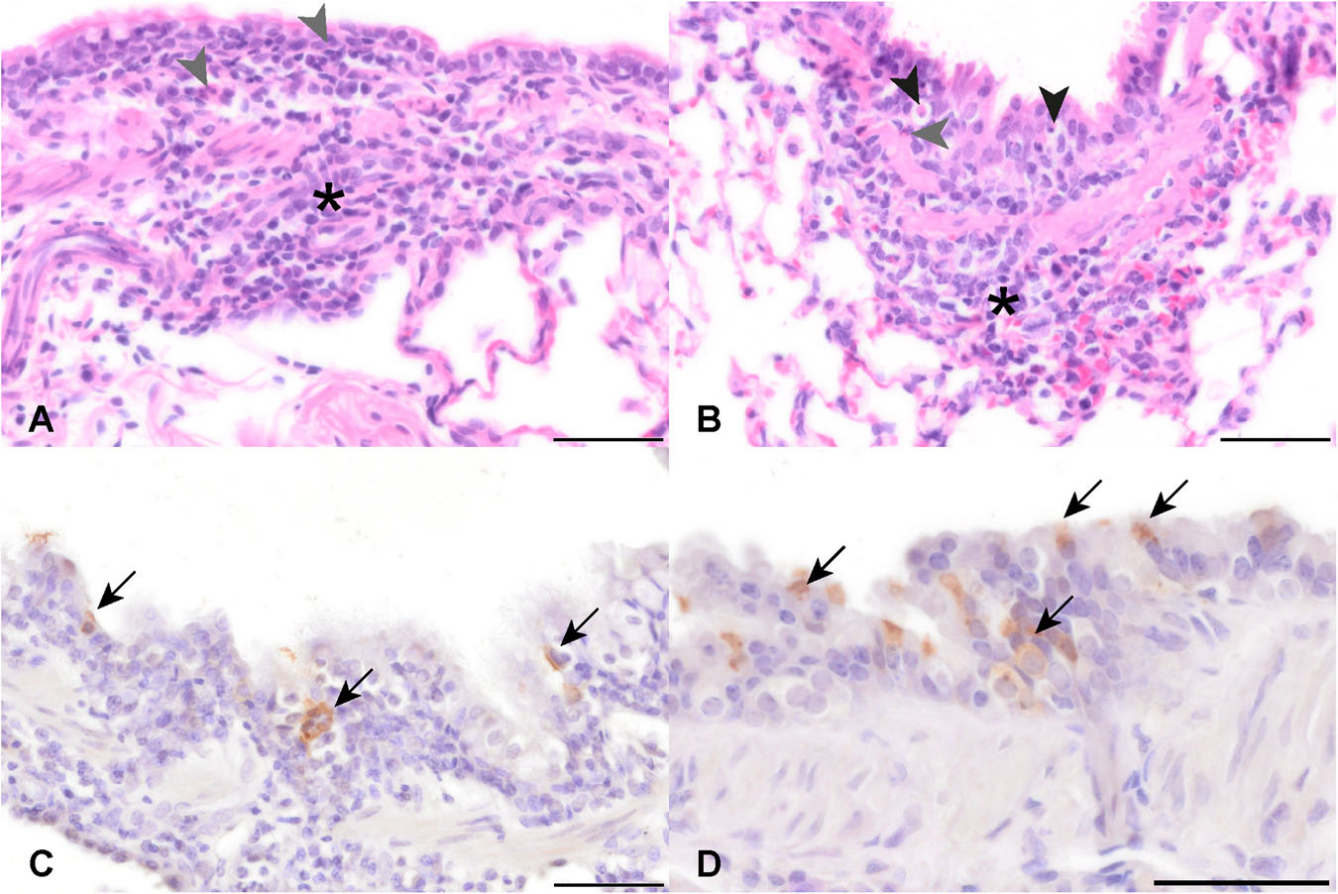
RSV infection of conductive airway mucosa. A multifocal infiltration of intra-epithelial (grey arrowheads) a moderate multifocal peribronchial mononuclear cell infiltrate (asterisk) is present within H&E strained sections of the bronchial and bronchiolar mucosa of ferrets (**A**) and hamsters (**B**). Multifocal respiratory epithelial cells in ferret (**C**) and hamsters (**D**) lung tissue are immunolabeled for RSV antigen by chromogenic DAB immunochemistry (arrow). Bars (**A**–**D**), 50 μm.

Lung lesions were evaluated as lesions of conductive airways and alveolar lesions. Lung changes were more pronounced in the ferrets than in the hamsters. Conductive airway lesions were characterized by vacuolation and loss of polarity of epithelial cells with varying degrees of a mainly mononuclear (lymphocytes, macrophages) and partly granulocytic infiltration. Single cell necrosis of epithelial cells was characterized by rounded hypereosinophilic epithelial cells with hyperchromatic and shrunken nuclei (Fig. 5A, B). Occasionally, there were eosinophils within the lamina propria and submucosa of affected airways. The peribronchial/perivascular infiltrate was mainly composed of mononuclear cells with low numbers of admixed granulocytes. Vascular endothelium was hypertrophic and pavementing of leukocytes was occasionally noted in both species. In the ferrets, mild (score 1) to moderate bronchitis and bronchiolitis (score 2) with moderate to marked (score 2 and 3) peribronchial/perivascular cuffing was detected in all animals. In one animal (animal #7), there was marked bronchiolitis (score 3) in one of the examined sections. In hamsters, conductive airway lesions were mainly defined by peribronchial/perivascular cuffing. Seven hamsters showed mild cuffing (score 1), whereas one animal each had a moderate (score 2) or marked cuffing (score 3). Bronchitis/bronchiolitis was detected in 5 animals (score 1: 3 animals; score 2: 2 animals). Alveolar lesions were characterized by intra-alveolar accumulation of macrophages and occasional granulocytes (Fig. S3). Occasionally, alveolar septae were variably hypercellular due to infiltration of mainly histiocytes and less frequent granulocytes. Pneumocyte type II hyperplasia was characterized by a cobblestone-like lining of hypertrophic type II pneumocytes along the alveolar septae. Additionally, varying numbers of intra-alveolar multinucleated syncytial cells were detected in the ferret, but not in the hamster lung. In eight ferrets, less than 25% of alveolar tissue was affected while in the ninth ferret (animal #7), more than 50% of alveolar parenchyma showed lesions. Inflammatory severity scores ranged from 1 to 3 with animal #7 also having the highest severity score. In the hamsters, mild alveolar lesions were detected in eight hamsters, the ninth hamster did not show alveolar lesions. In all affected hamsters, lesions extended into less than 25% of lung tissue. Seven of the hamsters had a severity score of 1, while in one histopathological examination yielded in a severity score of 2. In all ferrets and two hamsters, hyperplasia of type II pneumocytes was present. RSV antigen was detected within the bronchial and bronchiolar epithelium of seven ferrets and two hamsters. The conductive airways of the remaining animals yielded negative results (Fig. 5C, D). Alveolar immunolabeling for RSV antigen was only detectable in ferret #7 which also showed the most severe total lung score (13/15, 86.67% of maximal total score). No hamster showed alveolar immunoreactivity for RSV antigen.

## Discussion

In this study, we have assessed the hamster as an animal model for RSV infection in comparison to the established ferret model. The worldwide disease burden incurred by RSV infections of individuals from vulnerable populations and the rebound in case rates following the COVID-19 pandemic has resulted in an increased focus on implementing the targeted use of two newly approved vaccines and one new approved monoclonal antibody^7,8,18^. With this increased use of vaccines and therapeutics, there is also a need for increased epidemiological surveillance of sequence evolution in currently circulating RSV strains, particularly in antigenic epitopes in the F protein, which might lead to reduced efficacy of vaccines or therapeutics. In particular, there are indications that RSV-B strains are displaying higher rates of sequence changes in epitopes^19^, as illustrated by the failure of Suptavumab (REGN2222) in a Phase III clinical trial due to two amino acid changes in antigenic site V of the circulating RSV-B strain^20^. It is therefore crucial to complement in vitro assessment of contemporary RSV strains with readily accessible animal models susceptible to robust upper respiratory tract infection to enable better assessment of a range of parameters, such as antibody escape or antiviral drug resistance as a consequence of ongoing strain evolutions^21^.

High levels of viral replication were detected in the ferret throat and nasal swabs, with titers reaching 3 to 4 log10 TCID50/mL after 3 dpi. In contrast, hamsters had lower virus titers in throat swabs, often below the limit of detection, There was increasing viral copy numbers over time, indicative of ongoing virus replication, but this was not at detectable levels when the samples were titrated. Both species exhibited high titers and copy numbers in nasal turbinate tissue, demonstrating effective upper respiratory tract replication whereas replication in the lower respiratory tract was limited in both species. Limited replication of laboratory-adapted and older RSV-A strains in the respiratory tract of hamsters has been previously reported in the absence of overt macroscopic and histological evidence of pulmonary pathology^16^. A recent study by Levy et al. showed higher viral replication of a wt RSV in the hamster lung, while the recombinant vaccine strains also showed attenuated viral replication in the lung compared to the nasal turbinate, which is at least in part consistent with the results of this study^22^. A further area for investigation is the study of horizontal transmission between hamsters, which is a crucial aspect in establishing them as animal models for RSV. There are currently no studies investigating this aspect, but the possibility of horizontal transmission has already been demonstrated in the ferret model^23,24^. Therefore a similar study could be performed with hamsters. And to further evaluate hamster as animal model for RSV, a comparison study with cotton rats would be indicated.

In summary, hamsters appear to be a suitable model for studying RSV infection in the upper respiratory tract. Although ferrets displayed more severe systemic and pulmonary responses, hamsters showed t viral replication and pathological changes in the upper respiratory tract. Given that hamsters are cost-effective and easy to handle, this model may provide a viable alternative for in vivo RSV studies.

## Material and Methods

### Virus and cells

HEp2-cells were obtained from the American Type Culture Collection (ATCC, Manassas, USA), and were cultured in StableCell™ MEM (EMEM, Sigma-Aldrich, Merck KGaA, Darmstadt, Germany) with 10% fetal bovine serum (FBS) and 1% Penicillin/Streptomycin (P/S), and were used for virus growth and titration. A recombinant RSV strain (rRSV-A-0594) expressing EGFP based on a contemporary subtype A isolate was used for the in vivo infections and was grown and titrated as described previously^17^.

### Infection of ferrets and hamsters

Nine eight-month-old female ferrets (Euroferret, Denmark) and nine 10-week-old male Syrian hamsters (Janvier, France) were infected with 10^5^ TCID_50_ rRSV-A-0594-EGFP via the intranasal route (IN) using a dose volume of 0.3 mL in ferrets and a dose volume of 0.1 mL in hamsters. The inoculum was divided equally over both nostrils. The ferrets were also infected via the intratracheal route (IT) using a dose volume of 3.0 mL. Nasal and throat swabs of the ferrets were collected daily from days 1 to 6 post infection (pi). Due to the limited size of the nasal passages, nasal swabs from infected hamsters were not collected. Therefore, only throat swabs were collected from hamsters daily from days 1 to 6 pi. The swabs are placed immediately in virus transport medium (EMEM (Sigma-Aldrich) containing 2% HEPES (Sigma-Aldrich), 11% Pen/Strep (Sigma-Aldrich), 5% Amphotericin B (Sigma-Aldrich) and 2.5% BSA fraction V (35%) (Sigma-Aldrich)). They were then vortexed briefly and an aliquot was snap-frozen and stored at −80 °C for later RT-qPCR analysis. Hamsters and ferrets were euthanized on days 4, 5, and 6 pi (*n* = 3 per species, per time point). Images under UV light were taken of different skulls, lungs, and tracheas of the animals and tissue samples were collected. Lung and nasal turbinate tissue samples were weighed, homogenized, and clarified by low-speed centrifugation. The supernatant was collected for titration and an aliquot was snap-frozen and stored at −80 °C for later RT-qPCR analysis. Lung, nasal turbinate, and trachea tissue were collected and placed in 10% neutral formal saline for later histological examination and for immunohistochemistry. All animals were checked and weighed daily.

### Virus titration

Virus titrations of clinical samples were performed directly on the day of sample collection (days 1 to 6 pi for the swabs and days 4 to 6 pi for the tissue samples). In brief, quadruplicate, five-fold serial dilutions of samples were transferred to 96-well plates into which HEp-2 cell monolayers had been seeded one or two days previously and incubated for one hour at 37 °C. Inoculum was removed and cell monolayers were washed prior to the addition of DMEM (BioWest, Nuaillé, France) containing 1% L-Glutamine (Sigma-Aldrich), 1% Pen/Strep (Sigma-Aldrich), 1%FBS (Capricorn Scientific GmbH, Ebsdorfergrund, Germany), 1.1% Sodium Bicarbonate (Thermo Fisher Scientific, Eindhoven, The Netherlands) and 2% HEPES (Sigma-Aldrich). Cells were incubated for three or four days at 37 °C and 5% CO_2_. The presence of replication-competent virus was microscopically determined by the presence of EGFP fluorescence and viral titers were calculated using the Spearman–Karber method (converted to and reported as log_10_ TCID_50_/mL or /g). The lower limit of detection (LLOD) for titration of swab samples was 0.8 Log_10_ TCID_50_/mL. Virus titers were classified as undetectable when <0.8 Log_10_ TCID_50_/mL or detectable when ≥0.8 Log_10_ TCID_50_/mL. The LLOD for titration of tissue samples was variable, as it was dependent on the weight of the individual tissue sample.

### Reverse transcription quantitative real-time PCR (RT-qPCR)

Extraction of RNA from swab and tissue samples was performed using the NucleoMag VET kit (Macherey-Nagel GmbH & Co. KG, Düren, Germany). Following quantification of RNA samples using a Multiskan™ (Thermo Fisher Scientific), a Luna® One-Step RT-qPCR was performed using primers (RSV-A-0594_1181+, AGATCAACTTCT**A**TCATCCAGCAA; RSV-A-0594_1264-, TTCTGCACATCATAATTAGGAGT**G**TCAAT) and a probe (RSV-A-0594_1208+, FAM-**T**ACCATCCAACGGAGCACAGGAGA**C**-BHQ1) previously reported by van der Pol and colleagues^25^ which have been modified (altered nucleotides in bold) to take into account minor sequence evolution of contemporary RSV-A strains.

Following an initial 10-minute reverse transcriptase step at 55 °C, and incubation for one minute 95 °C, 45 cycles two step cycles were performed of 10 s at 95 °C and 30 s at 60 °C. The copy number of each sample was calculated using a standard curve (Fig. S1).

### Standard curve

An 84 bp amplicon generated in the RT-qPCR assay for RSV-A was cloned into the vector pMA-RQ by GeneArt (Thermo Fisher Scientific). RSV-A-0594 standard control: AGATCAACTTCTATCATCCAGCAAATATACCATCCAACGGAGCACAGGAGACAGCATTGACACTCCTAATTATGATGTGCAGAA. This plasmid was used for in vitro transcription using the HiScribe® T7 High Yield RNA Synthesis Kit. The resulting RNA was used to generate a standard curve. Based on the ct-values of the standard curve a linear regression was calculated, which was used to calculate the copy number.

### Histology

Tissues collected from each animal at the time of necropsy were fixed in 10% neutral-buffered formalin. Four sections from left ferret lung lobes (locations, see comment), one section from left hamster lung lobe and one section from the mid-trachea were dehydrated, embedded in paraffin, trimmed, and stained with hematoxylin-eosin according to routine protocols. Left nasal turbinates from ferrets and left paramedian longitudinal section of the hamster head were decalcified with ethylenediaminetetraacetic acid after fixation prior to embedding (Decalcifier soft, Carl Roth GmbH & Co. KG, Karlsruhe, Germany). Tissues were evaluated according to the following parameters, adapted from Stittelaar et al. ^15^: alveolitis severity, bronchitis/bronchiolitis severity, tracheitis severity and rhinitis severity: 0 = no inflammatory cells, 1 = few inflammatory cells, 2 = moderate number of inflammatory cells, 3 = many inflammatory cells; alveolitis extent: 0 = 0%, 1 = < 25%, 2 = 25–50%, 3 = > 50%; alveolar edema presence, alveolar hemorrhage presence, type II pneumocyte hyperplasia presence: 0 = no, 1 = yes. Extent of peribronchial/perivascular cuffing, 0 = none, 1 = 1–2 cells thick, 2 = 3–10 cells thick, 3 = > 10 cells thick. Bronchitis and bronchiolitis were scored as one value (conductive airway) in hamsters as they lack bronchial cartilage. As established, separated values were noted in ferrets^15^.

### Immunohistochemistry (IHC)

Sections were rehydrated and proteinase-induced antigen retrieval was performed for 15 min with proteinase K at 37 °C (pH 8.3). Sections were blocked with inactivated horse serum and incubated with a primary polyclonal goat anti-RSV antibody (1:1500, Cat. ab20745, Abcam Ltd, Cambridge, United Kingdom) or the respective concentration of goat serum (negative control, Suppl Figure S4) overnight at 4 °C. Subsequently, a biotinylated secondary horse anti-goat antibody (1:200, Cat. No. BA9500; Vector Laboratories Inc, Newark, California, United States) was added followed by Vectastain® Elite ABC kit (Vector Laboratories Inc.). Immunoreactivity was visualized with 3,3’ diaminobenzidine (Sigma Aldrich Chemie GmbH, Taufkirchen, Germany) and hematoxylin counterstain. Immunoreactivity in nasal mucosa (olfactory, respiratory), trachea, conductive airways and alveolar lining cells was scored semiquantitatively (0 = no labeled cells; 1 = < 25% labeled cells; 2 = 25–50% labeled cells; 3 = > 50% labeled cells). For visualization of lesions and immunoreactivity, slides were digitalized with an Olympus VS200 digital slide scanner and the respective OlyVIA software (Olympus Deutschland GmbH, Hamburg, Germany). Representative median lesions of hamsters and ferrets were selected for H&E images as well as randomly selected immunoreactive areas for RSV antigen detection.

## Supplementary information

Supplemental_4a193194-d765-4d92-8ac5-bad73eef58c9

## Data Availability

The datasets used and/or analyzed during the current study available from the corresponding author on reasonable request.
