# Supplementary material for: Syrian hamsters (Mesocricetus auratus) as an upper respiratory tract model for respiratory syncytial virus infection
Source: Npj Viruses. 2025 Jan 8;3:2. doi: 10.1038/s44298-024-00086-6 (PMC11721388; doi:10.1038/s44298-024-00086-6)

## Supplemental

**Figure S1.** Assessment of amplification efficiency for an RT-qPCR assay for RSV. Standard curves for internal template RNA. Mean Cq is plotted against mean input RNA copy numbers from seven replicates of 10-fold dilutions. The linear area of the plot is shown. Linear regression analysis for panels a to c was performed using Excel 2016.

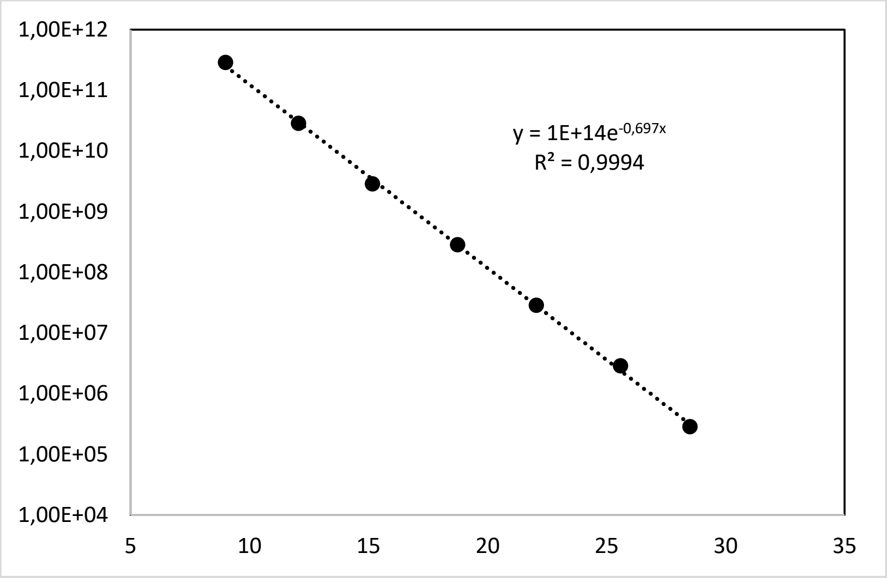

**Figure S2.** Average body weight change in ferrets and hamsters 4-6 dpi after infection with $10^5$ TCID50/dose rRSV-A-0594-EGFP virus. As body weight changes are transient, changes of up to 5% are considered normal and indicated by dashed lines.

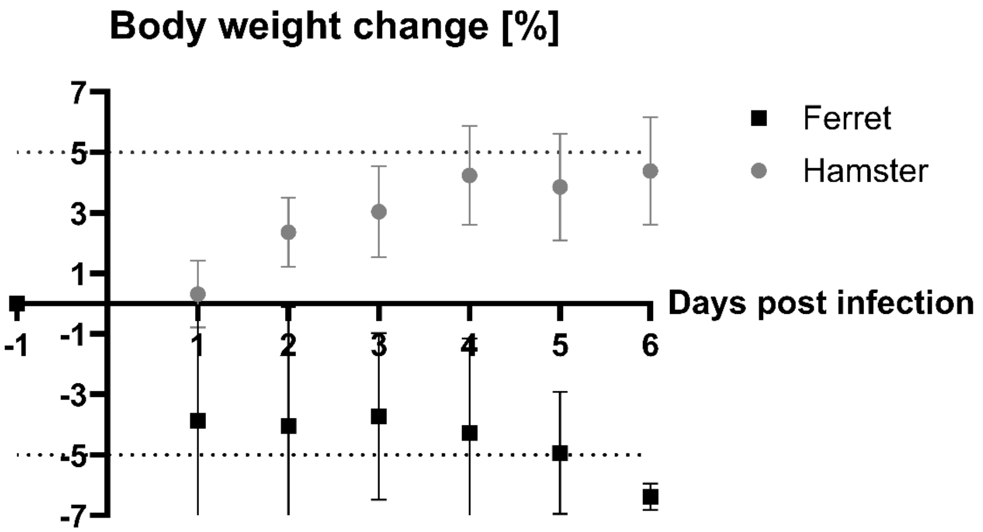

**Figure S3.** Alveolar lesions in H&E stained tissue sections from lungs from ferrets (A-C) and hamsters (D) infected with $10^5$ TCID50/dose rRSV-A-0594-EGFP. (A) Within the lower respiratory tract of the ferret lung, numerous foci (asterisk) of a mixed cellular infiltrate composed of neutrophils, macrophages and lymphocytes are present. Single necrotic cells can also be detected. (B) Some foci are mainly composed of primarily macrophages (asterisk) (C) Occasional multinucleated syncytial cells (arrows) are detectable (D) Alveoli in hamster lung tissue are sporadically filled with increased numbers of macrophages (asterisk). Bars (A-D), 50 µm.

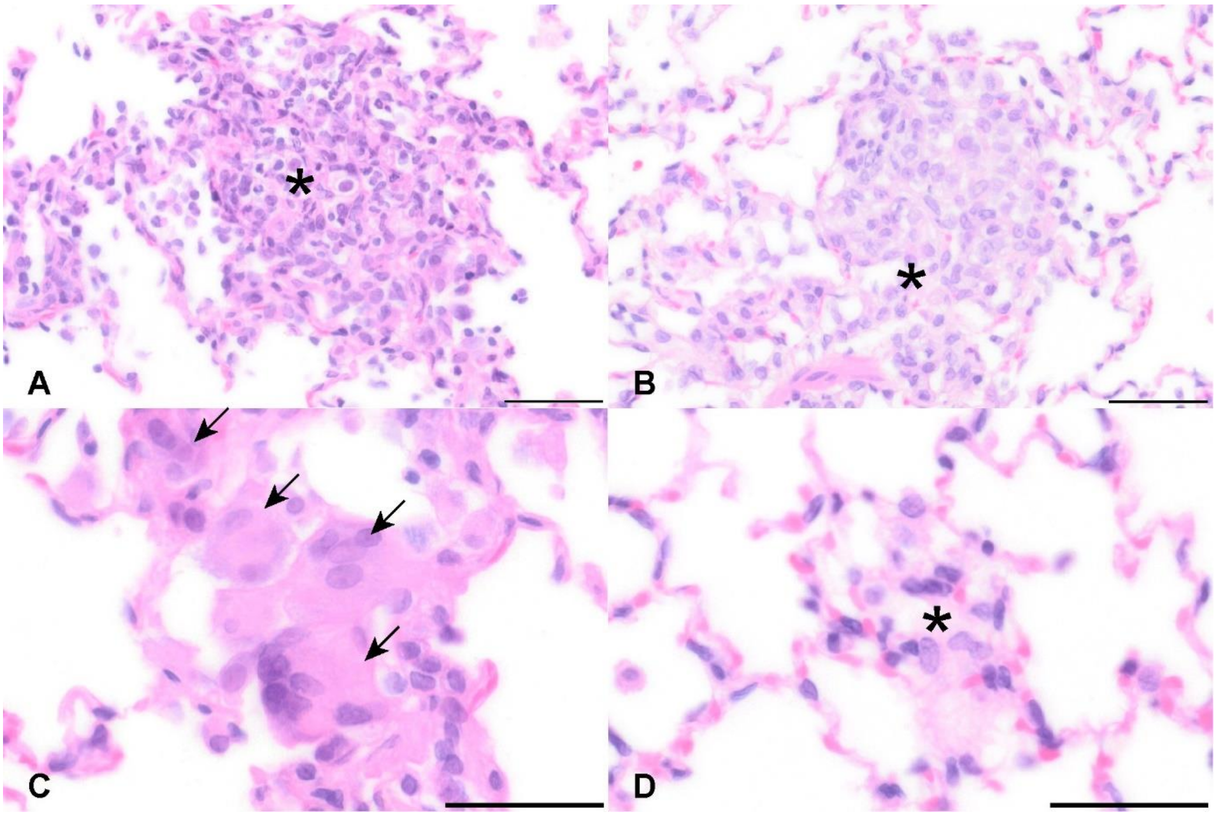

**Figure S4.** Immunohistochemistry negative controls. Replacement of the primary RSV antibody with goat serum led to absence of immunoreactivity in all examined tissues, no unspecific staining of goblet cells in the large airways of ferrets was detectable. Representative images of ferret (A) and hamster (B) nasal turbinates as well as ferret lung (C, D). Bars: A, B, D - 500µm; C - 100 µm.

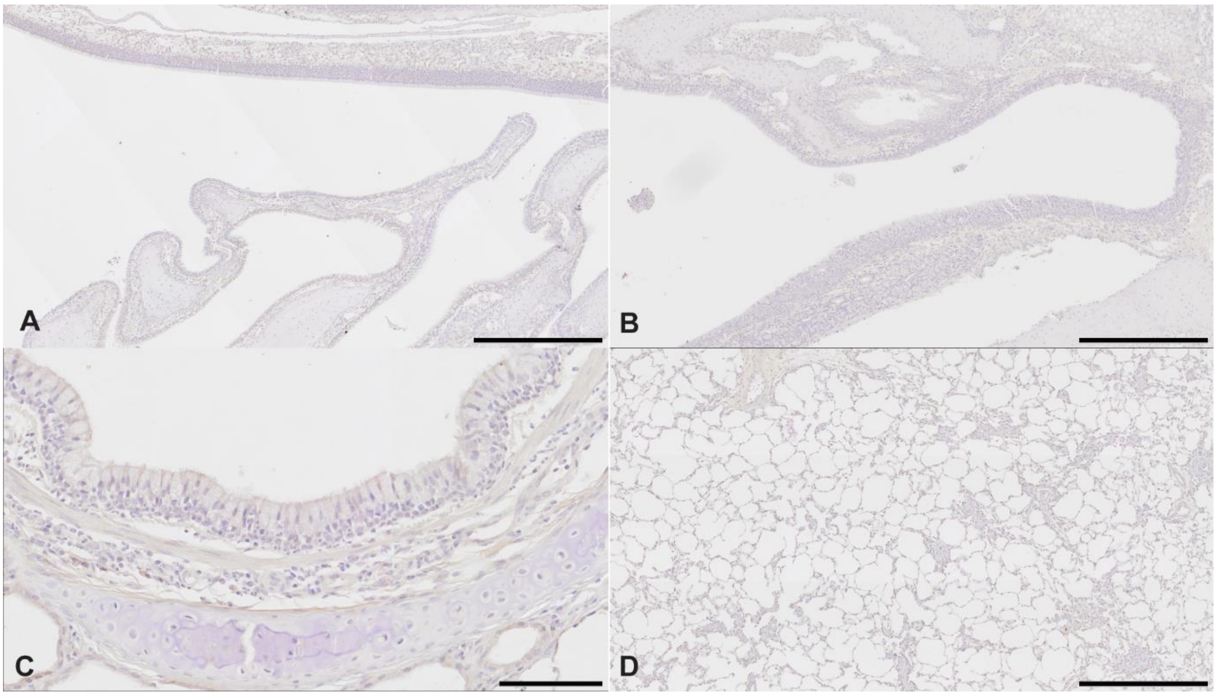

Supplement: Supplementary file 1 — Supplemental_4a193194-d765-4d92-8ac5-bad73eef58c9 [file 44298_2024_86_MOESM1_ESM.pdf]
